# Naso-Pharyngeal Lesions in Childhood

**Published:** 1902-09-13

**Authors:** 


### Hospital Clinics and Medical Progress.

### NASO-PHARYNGEAL LESIONS IN CHILDHOOD.

In the very interesting discussion which took place at the Manchester meeting of the British Medical Association, on the value of respiratory exercises in the naso-pharyngeal lesions of childhood, more attention was paid to the various theories as to the causation of the morbid conditions dealt with than to the particular kinds of exercises which are to be employed for their relief, and perhaps we may be allowed to take it for granted that the respiratory exercises alluded to have for their aim two main purposes—first, the establishment of complete respiration, and, second, the direction of a fair share of that respiration through the nose, so as to ensure the full and complete aëration of the nasal passages. Mr. Arbuthnot Lane opened the discussion by a paper in which he gave a full account of the serious and widespread consequences of defective nasal respiration. The primary factor which antedates any lengthy interference with the pressure exerted by the air on the walls of the naso-pharynx is, he says, a reduction of the amount of air which habitually passes in and out of the lungs, or in other words, is a diminution of the vital capacity of the individual. For some reason or another the child ceases to change a full volume of air during the respiratory process, the thoracic mechanism falling completely, or almost completely, into abeyance, and the diaphragm and abdominal muscles being alone made use of. The strength of the child and the nutrition of its tissues are reduced along with this diminution of the oxygenation of its blood, and thus the child's power of resistance to invading organisms, which find in the naso-pharynx their most accessible point of attack, is seriously interfered with. In consequence of these organisms obtaining a foothold in the naso-pharynx, the symptoms of what is popularly described as a cold in the head manifest themselves, and if the vigour of the child be not improved these organisms remain permanently in possession, and their presence helps still further to depreciate the energy and respiratory capacity of the sufferer. Thus we have initiated that imperfect aëration of the naso-pharynx and that lowered pressure—that negative pressure, as it is sometimes described—within the nasal passages and the upper part of the pharynx which is at the root of so many evil consequences. These consequences may shortly be stated as comprising the following conditions :—A narrowing of the alveolar arch. (1) An increase in the height of the palate along the middle line. (2) A diminution in the breadth and height of the nasal cavities. (3) Lateral compression of the anterior nares, the outer margins of which show no appreciable movement during respiration. (4) The mouth is kept habitually open, the upper lip covering only a portion of the incisors instead of projecting below their free margin. (5) The temporary teeth decay rapidly. This results partly from the collection of food and epithelium about the teeth and gums, and its drying and decomposition. Thus infective changes are produced which extend to the fauces and the tonsils. (6) The passage into the stomach of the infective organisms associated with the decomposition going on in the mouth leads to an unhealthy condition of the gastro-intestinal mucous membrane. (7) In association with the infection of the mucous membrane of the naso-pharynx there ensues an inflammatory condition of the lymphatic tissue of the upper part of the pharynx comprising the pharyngeal tonsil, which when inflamed constitutes what goes by the name of "adenoids." Various secondary infections of the lymphatic glands also result from this condition. (8) As a further result of this condition of the naso-pharynx the perfection of the auditory apparatus may be impaired or destroyed. (9) Various alterations in the anatomical relations of the bones of the face also result, which not only have a distinctly injurious æsthetic effect, but, by interfering with mastication, tend to produce and perpetuate various digestive troubles.

Now, although when adenoids have existed for some time, and are consequently associated with a considerable degree of imperfect development of the nasal cavities, their removal is called for in order to allow a free air-passage through the naso-pharynx, the primary root of the difficulty is the defective breathing, just as the primary root of the defective development of the bones of the face is a lack of the pressure exerted by the air in its passage to and fro through the naso-pharynx which is the chief factor in the development of that cavity and of the bones which surround it; and Mr. Lane maintains that all these troubles may be avoided altogether if proper measures be adopted from the first, that is, by proper attention to the aëration of the lungs, the ventilation of the naso-pharynx, and the proper performance of the functions of the body. This is generally admitted. The question of interest arises in regard to the course to be pursued when actual obstruction has developed, and in regard to this Dr. Scanes Spicer brought the discussion round to a scientific basis by insisting that obstructions in the naso-pharynx were not alone responsible for the evils which had been so well described by Mr. Lane. Negative pressure in the respiratory tract may, as he showed, arise also from nasal stenosis; and if Dr. Spicer is correct in his interpretation of the facts, such stenosis may be the very root even of the causation of adenoids. The teachings of modern pathology are, he said, that adenoids are the result of a deviation from the health of the normal adenoid tissue of the naso-pharynx; that their hyperplasia is the result of chronic hyperæmia; that this chronic hyperæmia is the result of a constant suction action on the walls of the nasal and naso-pharyngeal parts of the respiratory tract; and that nasal stenosis produces such a negative pressure as to account for this force of suction. "What other explanation could they tender as an alternative, or of higher probability, or so capable of explaining the associated conditions of the face and thorax, as this constantly acting force—tugging at the vascular walls seventeen times a minute, day and night, year in and year out, and enormously increased on exertion or forced breathing?" From all of which we may draw the moral that whatever are the benefits to be derived from respiratory exercises, the first step—the step which is necessary even to enable them to exert their full utility—is to remove the obstruction. If there exist in some cases mere functional flabbiness, well and good, let the respiratory exercises be used; while even in vigorous children conscious direction of the attention to the breathing function can only be beneficial if there be no obstruction. But if there be nasal stenosis (especially if structural), breathing exercises, says Dr. Spicer, can and do effect much harm, and waste invaluable time at a most critical period of life. Deformities of face, thorax, and spine, and deafness may be increased; the heart may give way, nervous symptoms may arise, and the feeble child may be quite exhausted by the energy necessary for the performance of these exercises under unsuitable conditions.

Every man tends nowadays to run his specialty as hard as he can, but no one can question the fact that Dr. Scanes Spicer stood on a good, firm, scientific foundation in insisting that if all the evil consequences commonly attributed to adenoids are due to abnormally distributed air-pressure, they can but be made worse by respiratory efforts made during the continuance of the obstruction upon which this abnormal distribution of air-pressure depends; while in regarding the adenoids themselves as an effect of stenosis elsewhere rather than as merely a result of inflammation he has much to say for his contention. The initial stenosis he believes from clinical observation to be usually either a common cold or a traumatism, and starting from this an ever-increasing vicious circle is formed unless the process be arrested. Selected and used with judgment after the rectification of structural impediments to breathing, respiratory exercises are then to be regarded as beneficial, but a most careful search for such obstruction must be made before they are undertaken, for if used ignorantly they are all-powerful to bring about the most disastrous consequences.

